# Supplementary material for: Anticancer Activity of Bitter Melon-Derived Vesicles Extract against Breast Cancer
Source: Cells. 2023 Mar 7;12(6):824. doi: 10.3390/cells12060824 (PMC10047160; doi:10.3390/cells12060824)
Supplement: Supplementary file 1 [file cells-12-00824-s001.zip › cells-2162868-supplementary.pdf]

Supplementary Materials for

Anticancer Activity of Bitter Melon-Derived Vesicles Extract against Breast Cancer

Ting Feng, Yilin Wan, Bin Dai * and Yanlei Liu *

Institute of Nano Biomedicine and Engineering, Shanghai Engineering Research Center for Intelligent Diagnosis and Treatment Instrument, Department of Instrument Science and Engineering, School of Electronic Information and Electrical Engineering, Shanghai Jiao Tong University, 800 Dongchuan RD, Shanghai 200240, China

* Correspondence: daibin@sjtu.edu.cn (B.D.); liuyanlei@sjtu.edu.cn (Y.L.)

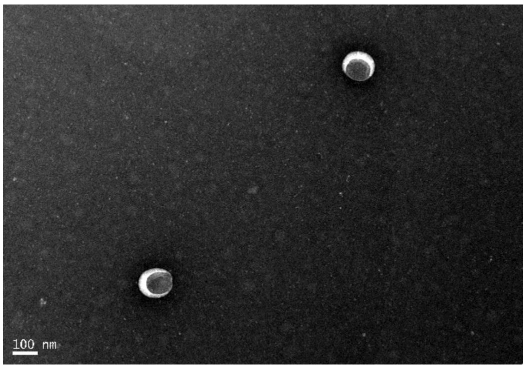

Figure S1. TEM characterization of BMVE (scale bar = 100 nm).

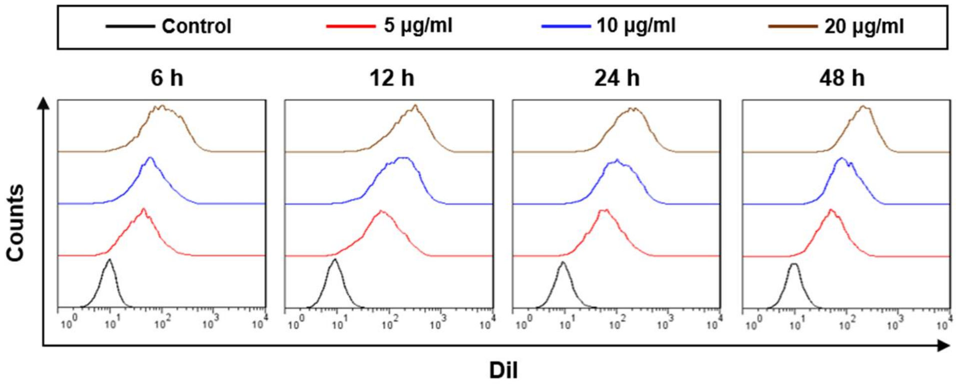

Figure S2. Internalization of BMVE by 4T1 cells. 4T1 cells were treated with 5, 10, and 20 μg/ml BMVE for 6, 12, 24, and 48 hours. The cells were collected and detected by flow cytometry to determine BMVE internalization by 4T1 cells.

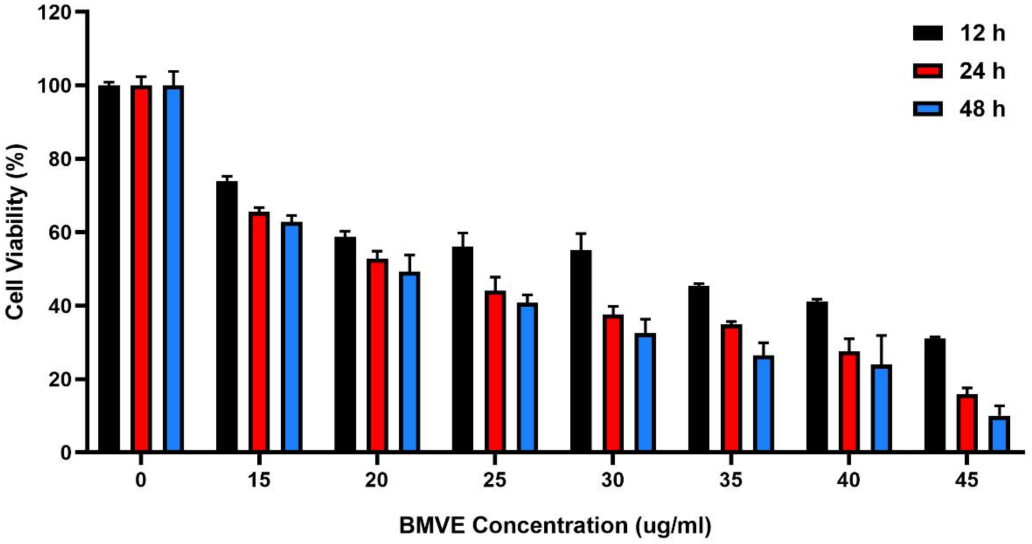

Figure S3. BMVE has anti-proliferation effects on MCF-7 cells. CCK-8 assay detected the viability of MCF-7 cells after treatment with varying concentrations of BMVE for 12, 24, and 48 hours.

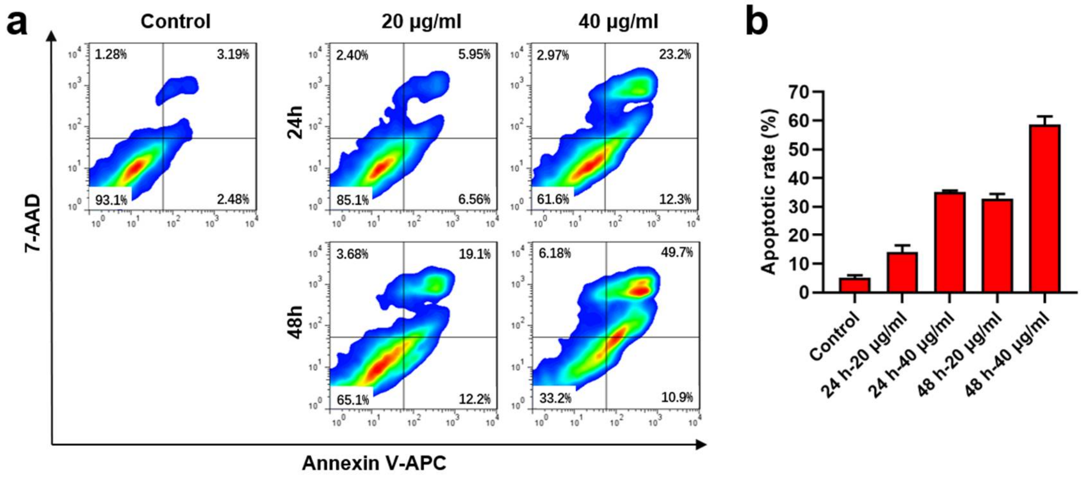

Figure S4. BMVE induces apoptosis in MCF-7 cells. (a) Representative images and (b) quantitative statistics of flow cytometry detection of 20 and 40 μg/ml BMVE induced apoptosis in MCF-7 cells at 24 and 48 hours.

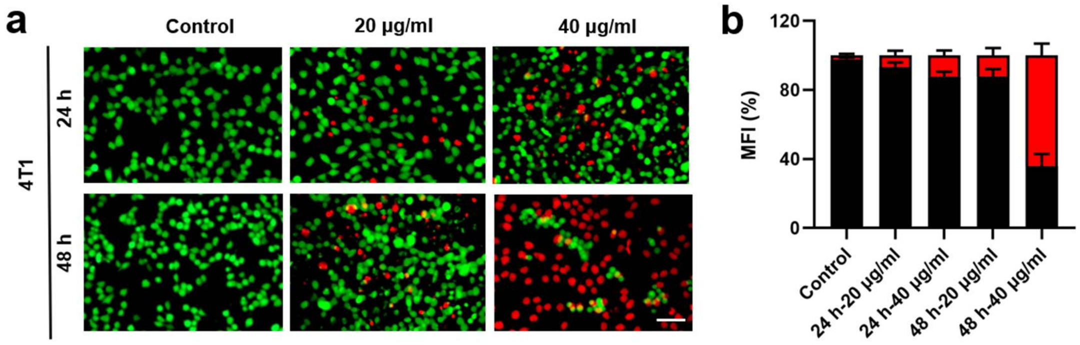

Figure S5. Calcein-AM/PI double staining assay of 4T1 cells. (a) After treating 4T1 cells with 20 and 40 μg/ml BMVE for 24 and 48 hours, respectively, the cells were double-stained with Calcein-AM/PI. Cell survival (green fluorescence) and cell death (red fluorescence) were observed using a fluorescence microscope (scale bar = 50 μm). (b) Quantitative statistics of fluorescence ratio of green and red about the double-stained cells (Black represents green fluorescent ratio; Red represents red fluorescence ratio).

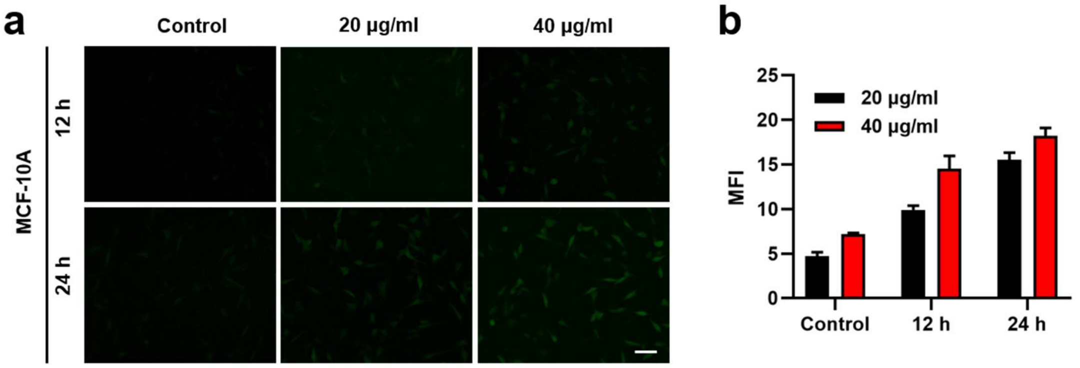

Figure S6. The ROS level in MCF-10A cells. (a) Representative images and (b) quantitative statistics of intracellular ROS level in MCF-10A cells treated with 0, 20, and 40 μg/ml BMVE at 12 and 24 hours, which was detected by DCFH-DA and observed by fluorescence microscopy (scale bar = 50 μm).

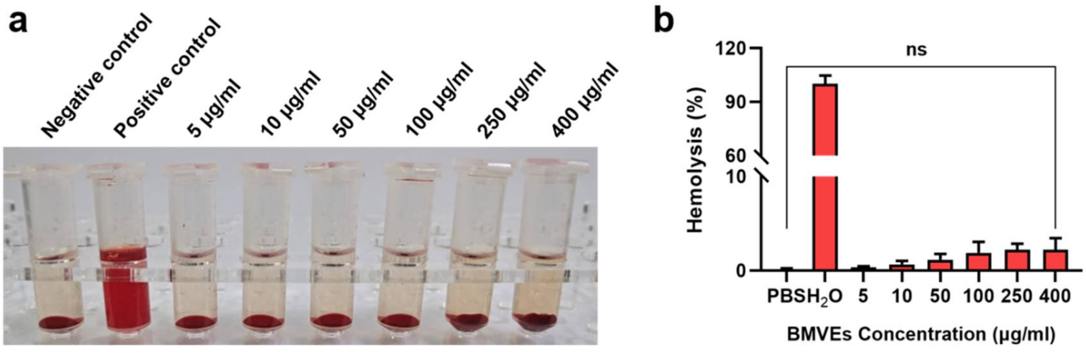

Figure S7. Hemolysis analysis of BMVE. (a) Representative images and (b) quantitative statistics of hemolysis by BMVE after centrifugation. Negative control is red blood cells (RBCs) in PBS and positive control is RBCs in water. The concentration range of BMVE in the experimental group was 5, 10, 50, 100, 250 and 400 μg/ml. mean ± SD, n=3
